# Yin Yang 1 (YY1) as a Central Node in Drug Resistance Pathways: Potential for Combination Strategies in Cancer Therapy

**DOI:** 10.3390/biom15081069

**Published:** 2025-07-24

**Authors:** Zhiyan Li, Xiang Jia, Ian Timothy Sembiring Meliala, Yanjun Li, Vivi Kasim

**Affiliations:** 1 College of Pharmacy and Biological Engineering, Chongqing University of Technology, Chongqing 400054, China; zhiyanli@stu.cqut.edu.cn (Z.L.); Jiaxiang@stu.cqut.edu.cn (X.J.); 2 Centre for Cellular Biology and Signalling, Zhejiang University-University of Edinburgh (ZJU-UoE) Institute, 718 East Haizhou Rd., Haining 314400, China; ian.20@intl.zju.edu.cn; 3 The Key Laboratory of Biorheological Science and Technology, Ministry of Education, College of Bioengineering, Chongqing University, Chongqing 400044, China

**Keywords:** Yin Yang 1, drug resistance, molecular mechanisms, cancer treatment, combination therapy

## Abstract

Tumor drug resistance, a major cause of treatment failure, involves complex multi-gene networks, remodeling of signaling pathways, and interactions with the tumor microenvironment. Yin Yang 1 (YY1) is a critical oncogene overexpressed in many tumors and mediates multiple tumor-related processes, such as cell proliferation, metabolic reprogramming, immune evasion, and drug resistance. Notably, YY1 drives resistance through multiple mechanisms, such as upregulation of drug efflux, maintenance of cancer stemness, enhancement of DNA repair capacity, modulation of the tumor microenvironment, and epithelial–mesenchymal transition, thereby positioning it as a pivotal regulator of drug resistance. This review examines the pivotal role of YY1 in resistance, elucidating its molecular mechanisms and clinical relevance. We demonstrate that YY1 inhibition could effectively reverse drug resistance and restore therapeutic sensitivity across various treatment modalities. Importantly, we highlight the promising potential of YY1-targeted strategies, particularly combined with anti-tumor agents, to overcome resistance barriers. Furthermore, we discuss critical translational considerations for advancing these combinatorial approaches into clinical practice.

## 1. Introduction

Yin Yang 1 (YY1) is a zinc-finger protein that belongs to the GLI-Krüppel family. In humans, *YY1* encodes a protein with 414 amino acids and four C2H2 zinc fingers domain, possessing activation and repression domains. The regulatory effect of YY1 on its target genes can be either positive or negative, which is determined by the specific binding context [1,2,3]. YY1 is ubiquitously expressed and capable of regulating approximately 7% of the total mammalian genome [4]. YY1 regulates transcription by binding to DNA sequences that most frequently contain the consensus core motifs 5′-CCAT-3′ and 5′-ACAT-3′. In addition to its transcriptional role, YY1 mediates post-translational modifications [5,6]. YY1 is a multifaceted protein with crucial functions in various biological processes, such as cell proliferation, chromatin remodeling, DNA synthesis, and embryogenesis [7,8,9,10,11]. Due to its critical role in biological procedures, dysregulation of YY1 is tightly bound to diseases, including cardiovascular diseases, nervous system diseases, metabolic disorders, and tumor diseases. Numerous studies have shown increased YY1 expression in various tumor cells, including breast, gastrointestinal, hepatocellular, and pancreatic cancers [12,13,14]. Extensive studies have demonstrated that *YY1*, as an oncogene, regulates many pathways in tumor cell growth, metabolic reprogramming, invasion, and chemotherapy resistance [15,16]. Mechanistically, YY1 activates oncogenic pathways, such as c-Myc and human epidermal growth factor receptor 2 (HER2), while suppressing those of tumor suppressors, including p53 as well as phosphatase and tensin homolog (PTEN), thereby promoting the cancer process. Furthermore, YY1 can rewire tumor cell metabolism by upregulating glycolytic enzymes and glutamine transporters and enhance tumor cells’ metastatic potential by reshaping the tumor microenvironment. In addition, YY1 facilitates tumor progression by regulating tumor immune response. YY1 reshapes the tumor immune microenvironment, including DNA methylation patterns, histone post-translational modifications, non-coding RNA networks, and core oncogenic signaling pathways, thereby promoting immune escape [17,18,19,20]. These findings position YY1 as a central node in cancer signaling networks, where it coordinately regulates uncontrolled tumor cell proliferation, metastasis, immune escape, and survival pathways, demonstrating the multifaceted programs driving tumor progression.

Despite being the therapeutic backbone in oncology, conventional and targeted anticancer agents universally face the formidable challenge of acquired resistance [21]. Tumor cells’ remarkable capacity for evolutionary adaptation systematically undermines treatment efficacy, invariably leading to either explosive cancer recurrence or accelerated malignant progression, representing the principal challenge to achieving durable remissions and improved survival [21,22]. Drug resistance is a primary cause of treatment non-response in oncology. Resistance arises through multiple molecular mechanisms, which collectively enable tumor cells to evade therapeutic pressures and sustain malignant progression [23,24]. Primary resistance describes tumors’ innate insensitivity to therapies due to their genetic and molecular features, reducing treatment efficacy from the outset [24]. Acquired resistance arises through tumors’ evolution under therapy, mediated by mechanisms like somatic mutations, signaling pathway dysregulation, increased drug efflux, and defective cell death in tumor cells [23,25].

Notably, numerous studies have shown that YY1 drives drug resistance development in tumors [26]. Mechanistically, YY1 mediates resistance through various mechanisms, including dysregulated signaling pathways, such as DNA repair modulation, drug efflux activation, cancer stemness maintenance, and apoptosis regulation. Moreover, preclinical models have shown that genetic or pharmacological inhibition of YY1 sensitizes resistant tumor cells to conventional treatments, highlighting its potential as a biomarker for predicting therapy and a treatment target for overcoming drug resistance [27,28,29].

In the present review, we highlight the function of YY1 in driving drug resistance in tumor cells, encompassing its regulation of survival pathways. We also discuss YY1 as a potential biomarker of drug resistance and its therapeutic potential for overcoming therapy resistance, with a focus on recent advances in pharmacological inhibition and potential combination strategies with conventional anticancer therapies.

## 2. The Function of YY1 in Tumor Drug Resistance Mechanisms

Despite remarkable advances in cancer therapeutics, resistance still is a critical challenge in cancer therapy, acting as the primary cause of treatment failure in most poor prognosis and survival rates. Drug resistance is associated with molecular changes promoting adaptive mechanisms. Elucidating the mechanisms underlying cancer drug resistance is therefore of paramount importance for improving therapeutic outcomes [25,30]. YY1 drives drug resistance through various pathways, including DNA repair signaling, anti-apoptosis processes, and stemness maintenance progress. A number of studies have shown that YY1 plays crucial roles in cancer drug resistance.

### 2.1. YY1 Enhances Drug Resistance Through Upregulation of Drug Efflux

**Figure 1:**
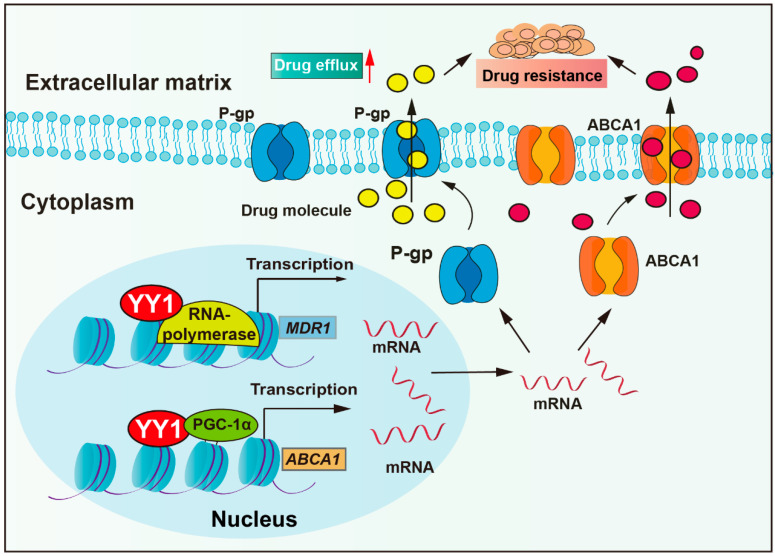
YY1 enhances drug resistance through upregulation of drug efflux. YY1 promotes drug excretion through cells by increasing the expression of P-gp and ABCA, leading to drug resistance. Arrows are used to show activation; arrows terminated with a bar represent inhibition.

Anti-tumor drugs require sufficient cellular concentrations to achieve therapeutic efficacy. However, tumor cells could actively reduce intracellular drug concentration by overexpressing pump proteins, thereby enhancing drug efflux. Drug transporter mediated efflux, particularly by ATP-binding cassette (ABC) proteins, is the most clinically significant drug efflux mechanism. ABC transporters utilize ATP-derived energy to drive the active translocation of various substrates. Numerous studies have unequivocally identified ABC transporters as pivotal players in mediating cancer multidrug resistance (MDR) development, such as P-glycoprotein (P-gp, also named MDR1), multidrug resistance-associated protein 1 (MRP1), and breast cancer resistance protein (BCRP) [31]. P-gp, encoded by the multidrug resistance-1 (*MDR1*) gene, is a central member of the ABCB subfamily within the ABC transporter family. P-gp actively extrudes therapeutic agents and is overexpressed in most cancers (colorectal cancer, breast cancer, glioma, lung cancer, and others), and it has been confirmed to correlate with clinical drug resistance [32]. Studies have revealed that YY1 binds to the promoter of the *MDR1* gene, enhancing transcriptional activity and upregulating P-gp protein expression, thereby reducing cellular sensitivity to multiple chemotherapeutic agents, including vincristine, cytarabine, and adriamycin (Figure 1) [33,34,35]. Moreover, clinical studies show that YY1 correlates positively with P-gp expression in acute lymphoblastic leukemia (ALL), with high YY1 expression patients exhibiting significantly lower 5-year overall survival rates [34,36]. In addition, a study demonstrated that the bendamustine hydrochloride (BH) resistant mantle cell lymphoma (MCL)-derived subline KPUM-YY1R showed significant upregulation of *MDR1* mRNA and cross-resistance to vincristine and cytarabine. Moreover, the restoration of BH sensitivity in KPUM-YY1R cells through P-gp inhibition confirmed that YY1 drives chemoresistance in tumor cells by upregulating *MDR1* gene expression and amplifying drug efflux capacity [33]. YY1 cooperates with transcriptional coactivator PGC-1α and then enhances transcriptional activity of the ATP-binding cassette transporter 1 (*ABCA1*) gene, resulting in elevated ABCA1 expression levels, leading to insensitivity of colorectal cancer cells to isoliquiritigenin and tumor metastasis [37].

In addition to drug transporters, studies have reported that exosomes also actively contribute to enhanced drug efflux. Emerging evidence indicates that the phospholipid composition of exosome membranes and internal lumen facilitates the binding and sequestration of hydrophobic drugs, such as docetaxel, resulting in reduced intracellular drug accumulation [38,39]. YY1 promotes the secretion of exosomes to promote prostate cancer progression, and YY1 upregulates polycom, also named pentraxin domain containing 1 (SVEP1), in cancer-associated fibroblasts (CAFs), which secrete *miR-146a-5p* and increase urothelial bladder cancer resistance [7,40,41,42]. These studies demonstrate that YY1 enhances tumor drug efflux and promotes multidrug resistance by regulating ABC transporters and promoting exosome-mediated drug efflux.

### 2.2. YY1 as a Critical Regulator of Cell Death Pathways in Cancer Drug Resistance

Cell death acts as a crucial factor in regulating impaired cells during developmental stages and is controlled by multiple signaling pathways. Nonetheless, its dysregulation may lead to tumor progression and therapeutic resistance [43]. Tumor cells can evade apoptosis by inhibiting the pro-apoptotic signal or upregulating the anti-apoptotic signal. This enables them to resist chemotherapy-induced cell death and contributes to therapeutic failure. The key mechanisms involved in apoptotic inhibition include overexpression of anti-apoptotic proteins, BCL-2, BCL-XL, and MCL-1, or downregulation of pro-apoptotic proteins (such as p53 and BAX) [44,45,46].

**Figure 2:**
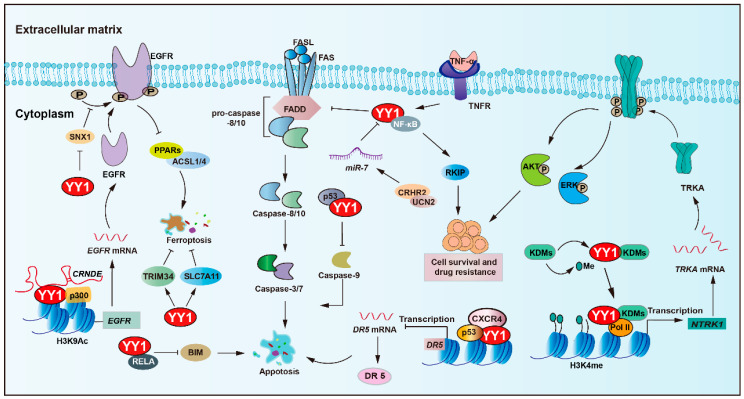
YY1 as a key regulator of cell death pathways in tumor drug resistance. YY1 promotes tumor cell survival and drug resistance through multifaceted anti-apoptotic mechanisms, including engagement in the NF-κB/SNAIl/YY1/RAF regulatory loop, upregulation of MCL-1 alongside inhibition of caspases-3, -7, and -9, and suppression of the death receptor pathway via p53/YY1-mediated downregulation of DR5 and direct inhibition of FAS expression. Additionally, YY1 inhibits BIM by forming a complex with the NF-κB subunit RELA, confers resistance to ferroptosis by inhibiting the PPARs-ACSL1/4 signaling axis and upregulating TRIM34 and amplifies EGFR signaling through YY1-mediated inhibition of SNX1 under nutrient stress. Collectively, these mechanisms enable YY1 to drive therapeutic resistance by robustly promoting anti-apoptotic responses in tumor cells. Arrows are used to show activation; arrows terminated with a bar represent inhibition.

YY1 plays a central role in regulating apoptosis and mediating resistance to anticancer therapies (Figure 2 and Table 1). An important pathway involving YY1 is the NF-κB/YY1/ RKIP regulatory axis, which has been shown to significantly contribute to tumor cell resistance against both chemotherapy and immunotherapy-induced cytotoxicity [47,48,49]. For instance, YY1 upregulates the expression of MCL-1, while concurrently downregulating pro-apoptotic caspases-3, -7, and -9 [43,50]. Such a dual regulatory mechanism promotes cancer cell survival and contributes to therapy resistance, potentially in a p53-dependent manner. Simultaneously, obatoclax-induced death receptor 5 (DR5) downregulation via YY1 and NF-κB activation concurrently promotes MCL-1, further influencing sensitivity to TRAIL-based therapies [51,52,53]. Additionally, YY1 cooperates with p53 to transcriptionally suppress DR5 expression upon chemokine receptor (CXCR4) activation, thereby promoting chemoresistance and tumor progression in solid tumors [54].

Furthermore, YY1 suppresses the expression of *BIM*, a pro-apoptotic gene, by forming a transcriptional repressor complex with the NF-κB subunit (RELA), further promoting resistance to programmed cell death [44]. Recent findings have also identified corticotropin-releasing hormone receptor 2 and its ligand urocortin-2 as modulators of colorectal cancer (CRC) cell sensitivity to FAS/FASL-induced apoptosis. Restoration of CRHR2/UCN2 signaling leads to *miR-7* overexpression and decreased YY1 expression, thereby resensitizing tumor cells to FAS/FASL-mediated cell apoptosis [55].

The tropomyosin receptor kinase A (TRKA), encoded by the neurotrophic tropomyosin receptor kinase 1 (*NTRK1*) gene, has been implicated in imatinib resistance. Mechanistically, YY1 recruits histone lysine demethylase 6A (KDM6A) to the *NTRK1* promoter, leading to upregulation of TRKA expression and activation of downstream survival pathways that confer resistance to imatinib [56]. Another histone demethylase, lysine demethylase 5C (KDM5C), which specifically demethylates H3K4, exhibits context-dependent oncogenic or tumor suppressor roles. Notably, YY1 and KDM5C form a synthetic lethal pair. KDM5C can recruit YY1 to the promoters of genes associated with cell cycle control and apoptotic processes, which promote drug resistance [57]. 

Additionally, non-coding RNA networks play a pivotal role in YY1-mediated anti-apoptotic regulation in tumors. The lncRNA *CRNDE* enhanced p300/YY1 binding to the *EGFR* promoter, leading to EGFR upregulation and resulting in enhanced tumor cell proliferation and sorafenib resistance [58]. In ovarian cancer, YY1 induces the transcription of *PART1*, enhances resistance to cisplatin, and inhibits apoptosis by modulating the *miR-512-3p*/chromatin accessibility complex subunit 1 (CHRAC1) axis [59]. Moreover, YY1 transcriptionally activates *miR-135b*, which directly suppresses the core circadian regulator BMAL1, to target and repress the circadian core gene *BMAL1*, thereby forming a YY1/*miR-135b*/*BMAL1* axis, inhibiting tumor cell apoptosis, and promoting gemcitabine resistance in pancreatic cancer [60].

Ferroptosis is a type of regulated cell death that relies on iron and is marked by the accumulation of lipid peroxides. A study showed that YY1 activates TRIM34 transcription, contributing to TRIM34 overexpression, thereby suppressing ferroptosis and promoting cell proliferation and migration in HCC [61]. Moreover, YY1 acts as a pivotal regulator in oxymatrine-induced liver cancer ferroptosis by modulating the silent information regulator 1 (SIRT1)/YY1/GPX4 signaling axis [62]. In addition, YY1 could bind to the solute carrier family 7-member 11 (SLC7A11) promoter and upregulate its transcription, which sustains cystine uptake and glutathione synthesis, ultimately conferring insensitivity to ferroptosis-inducing agents erastin and RSL-3 [63].

Another key regulator linked to YY1-mediated survival is sorting nexin 1 (SNX1), a protein involved in EGFR trafficking and degradation. SNX1 deficiency enhances EGFR phosphorylation, thereby increasing resistance to gefitinib. Under a nutrient-deficient environment, YY1 becomes activated, represses SNX1 expression, and consequently activates EGFR signaling. This cascade also inhibits the PPARs-ACSL1/4-mediated ferroptosis pathway, facilitating colorectal cancer cell survival and resistance to 5-FU [64].

Collectively, these findings underscore the central role of YY1 as a pivotal transcriptional regulator that bridges apoptotic resistance, cancer progression, and treatment failure. Targeting YY1 and its regulatory network represents a promising strategy for enhancing the efficacy of anticancer therapies.

### 2.3. YY1 Drives Drug Resistance by Regulating Cancer Stemness

**Figure 3:**
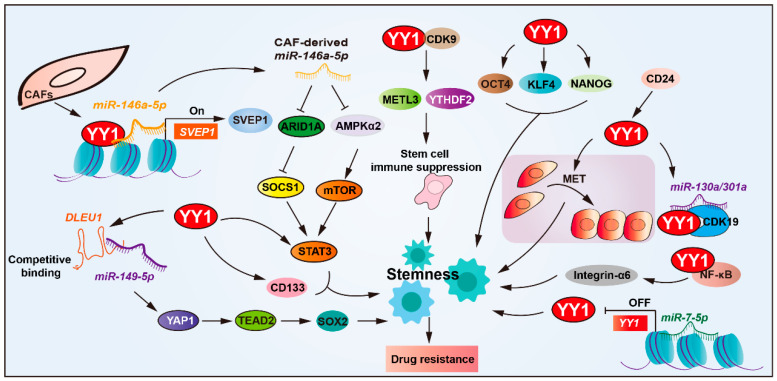
YY1 drives drug resistance by regulating cancer stemness. YY1 promotes tumor drug resistance by enhancing cancer stem cell (CSC) properties, directly upregulating CSC markers (CD133, STAT3, and integrin-α6). Through NF-κB/PI3K/AKT signaling, it forms regulatory loops with SOX2, OCT4, and BMI1 to reinforce CSC traits. The microRNA network modulates this process; *miR-146a-5p* enhances YY1 recruitment to upregulate SVEP1 in CAFs for CSC amplification, while *miR-7-5p* suppresses YY1 to reduce stemness. CD24 activates the YY1-*miR-130a*/*301a*-CDK19 axis to maintain CSC quiescence, and the YY1–CDK9 complex diminishes tumor immunogenicity, collectively driving CSC-mediated drug resistance. Arrows are used to show activation; arrows terminated with a bar represent inhibition.

Cancer stem cells (CSCs) drive tumor initiation, progression, and recurrence [65]. CSCs are the triggers of tumorigenesis and the initiators of tumor treatment resistance [66,67]. Among the transcriptional regulators involved, YY1 has emerged as a modulator of CSC-associated phenotypes and drug resistance across multiple cancer types (Figure 3 and Table 2).

YY1 overexpression exerts a positive regulatory effect on CSC stemness and drug resistance [7,68,69,70]. Studies have revealed that YY1 upregulates the expression of CSC markers’ (such as CD133, STAT3, and integrin-α6) expression, thereby promoting the resistance of glioblastoma cells to cisplatin and temozolomide. Deletion in lymphocytic leukemia 1 (DLEU1) enhances cancer stemness by upgrading upregulating stem cell markers (SOX2, OCT4, NANOG, and KLF4) in cholangiocarcinoma. YY1-mediated DLEU1 activation promotes resistance to cisplatin and gemcitabine, establishing a YY1/DLEU1/stemness axis in chemoresistance [71].

YY1 is also involved in modulating TME to support CSC-mediated resistance. In bladder cancer, the extracellular matrix protein SVEP1, a matrix-type CAF marker, has been implicated in chemoresistance. Mechanistically, *miR-146a-5p* enhances YY1 recruitment to upregulate SVEP1 expression in CAFs and enhances CSCs’ properties, and depletion of either *miR-146a-5p* or SVEP1 significantly sensitized urothelial bladder carcinoma (UBC) cells to gemcitabine–cisplatin combination therapy both in vitro and in vivo [7,72].

Post-transcriptional regulation of YY1 also influences stemness and therapy resistance. YY1 downregulated by *miR-7-5p* via direct 3′UTR targeting reduces stemness and enhances temozolomide sensitivity in glioblastoma, positioning the *miR-7-5p*/YY1 axis as a key regulator of chemoresistance. In ovarian cancer, YY1 expression is upregulated by CD24, a surface marker associated with CSC phenotypes and malignant progression. CD24-induced YY1 promotes mesenchymal-to-epithelial transition (MET), a process linked to enhanced stemness and platinum-based chemoresistance [73,74]. Furthermore, CD24 facilitates aberrant activation of the YY1-*miR-130a/301a*-CDK19 regulatory axis, promoting ovarian CSCs’ quiescence and platinum-based drug resistance [75].

YY1 also interacts with core stemness transcription factors, such as SOX2, OCT4, and BMI1. Together, they form part of a regulatory circuit involving NF-κB/PI3K/AKT signaling that reinforces CSCs’ maintenance and cisplatin resistance [76]. YY1 also interacts with CDK9 in glioblastoma stem cells and maintains stemness. Moreover, YY1–CDK9 complexes downregulate the immune response of tumor cells via NF-κB signaling, thereby enhancing resistance to immunotherapy in glioblastoma [77].

In summary, YY1 is a central transcriptional regulator of CSC biology and therapy resistance. It operates through direct transcriptional activation of stemness genes, modulation of tumor–stromal interactions, engagement with non-coding RNAs, and integration into broader oncogenic signaling pathways. Elucidating YY1’s role in maintaining CSCs and mediating resistance mechanisms provides promising avenues for enhanced cancer therapy.

**Table 2:** The stemness-maintaining function of YY1 contributes to drug resistance.

Molecular Pathway	Target Gene or Pathway	Regulation	Resistance Type	Refs
YY1/CD133	STAT3 and integrin-α6	Increases the CSC markers	Resistance to cisplatin and temozolomide	[78]
YY1/DLEU1	SOX2, OCT4, NANOG, KLF4	Actives DLEU1	Resistance to cisplatin and gemcitabine	[71]
*MiR-146a-5p*/YY1/SVEP1	SVEP1	*MiR-146a-5p* enhances the recruitment of YY1 and upregulates SVEP1	Resistance to the combination therapy of gemcitabine and cisplatin	[72]
*MiR-7-5p*/YY1	YY1’ 3′UTR	Downregulates YY1 by binding to 3′UTR of YY1	Resistance to temozolomide	[79]
CD24/YY1	YY1	CD24 upregulates YY1 and promotes mesenchymal– epithelial transition	Resistance to platinum drugs	[73]
YY1-*miR-130a/301a*-CDK19	YY1-*miR-130a/301a*-CDK19	CD24 promotes abnormal activation of the YY1-*miR-130a/301a*-CDK19 regulatory axis	Resistance to platinum drugs	[75]
YY1 with SOX2, OCT4, BMI1	NF-κB/PI3K/AKT	Strengthens the maintenance of CSCs	Resistant to cisplatin	[76]
YY1–CDK9 complexes	NF-κB	The complex downregulates the immune response of tumor cells	Resistant to immunotherapy	[77]

### 2.4. YY1-Mediated DNA Repair Pathways in Drug Resistance

Multiple anti-tumor agents, including 5-FU, platinum drugs, and temozolomide, achieve anti-tumor efficacy by inducing DNA damage to trigger tumor cell death [80]. However, tumor cells respond to DNA damage through coordinated signaling pathways, such as homologous recombination, non-homologous end joining, mismatch repair, and base excision repair pathways, to ensure survival. Consequently, enhanced DNA repair capacity constitutes a critical determinant of chemotherapeutic resistance in tumor cells [81,82].

RAD51 recombinase, an ATPase, assembles into a nucleoprotein filament on single-stranded DNA, which is a crucial player in the HR process, including searching for homologous sequences and strand exchange. YY1 binds to guanine quadruplexes’ (G4s) structures, which restricts RAD51 access and thereby shields excessive HR activity, ultimately protecting genomic stability [83]. YY1 also upregulates various DNA-repair-related proteins, including PTEN and RAD51. YY1 reduces the phosphorylation of PTEN by inhibiting the activity of protein phosphatase 2A (PP2A), thus ensuring the fidelity of the DNA repair process [84]. Moreover, the shieldin complex shields double-strand DNA breaks (DSBs) from nucleolytic resection. YY1 inhibits SHLD1 transcription, thereby leading cross-resistance to poly ADP-ribose polymerase (PARP) inhibitor and cisplatin in breast cancer susceptibility gene 1 (*BRCA1*)-deficient ovarian cancer cells [85]. In addition, bromodomain and extraterritorial domain family factors modulate the expression of several genes related to carcinogenesis. It is overexpressed in many types of cancer and associated with the resistance of tumor cells to chemotherapeutic drugs [86,87]. YY1 recruits BETs (including BRD2, BRD4) to bind to cis-regulatory elements of transcription factor 7 like 2 (TCF7L2), thereby regulating the expression of TCF7L2, cooperatively activating tumor cell DNA repair capacity and driving therapeutic resistance [88,89].

Furthermore, YY1 can regulate DNA repair by affecting the expression of related enzymes. PARP1 plays an important role in DNA repair processes. YY1 binds to the PARP1 promoter and then drives its expression; more importantly, the YY1–PARP1 axis highlights the potential of DNA repair and promotes resistance [90,91,92]. In addition, the elevated expression of flap endonuclease 1 (FEN1) correlates with drug resistance and patient survival in breast cancer. Studies demonstrate that YY1 functions as a transcriptional repressor of FEN1, and YY1 dissociates from the FEN1 promoter to increase its expression under DNA-damaging agent (mitomycin and taxol) treatment, consequently promoting resistance to drugs in breast cancer cells [93,94,95]. Similarly, aldo-keto reductase family 1 member C3 (AKR1C3) regulates cell cycle G2 arrest and the expression of RAD51, which is involved in the mechanism of DNA damage repair ability. YY1 upregulates AKR1C3 and the hedgehog axis to enhance lenalidomide (LEN) resistance in multiple myeloma cells [96,97].

**Figure 4:**
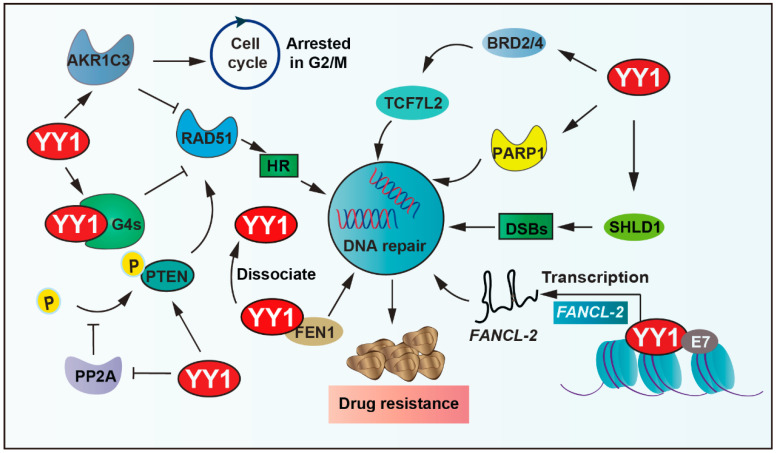
YY1-mediated DNA repair pathways in drug resistance. YY1 promotes DNA repair via transcriptional activation of SHLD1/PARP1, inhibiting PP2A-mediated dephosphorylation of PTEN, binding guanine quadruplexes (G4s) to restrict RAD51-dependent homologous recombination, and activating AKR1C3 and Hedgehog signaling, and recruits BRD2/4 to cis-regulatory elements of TCF7L2 to modulate its expression. These mechanisms enhance DNA repair and enable evasion of DNA-damage-induced cell death, establishing YY1 as a key mediator of therapeutic resistance. Arrows are used to show activation; arrows terminated with a bar represent inhibition.

Additionally, YY1 upregulates the expression of *lnc-FANCI-2* through interaction with a conserved region 3 (CR3) of E7 (human papillomaviruses oncoprotein) core motif and transactivates the promoter of *lnc-FANCI-2*, which is expressed from a genomic locus adjacent to the *FANCI* gene encoding an important DNA repair factor in in vivo and in vitro tumor cells [98]. YY1 further binds directly to the miR-140 promoter, which leads to decreased miR-140 expression, and then directly associates with its 3′UTR to inhibit flap endonuclease 1 (*FEN1*), which results in impaired DNA repair and reduced doxorubicin resistance [94]. In summary, YY1 drives tumor cell resistance by promoting DNA damage repair (Figure 4).

### 2.5. YY1 Orchestrates TME Remodeling to Drive Drug Resistance

The tumor microenvironment (TME) is a complicated system that includes the effects of the metabolic microenvironment, the immune microenvironment, the extracellular matrix, and angiogenesis [99,100]. The TME plays a crucial role in drug resistance development, which is a promising and strategic target for tumor therapies [101].

Due to the inadequacy of the vascular system, hypoxia and nutrient deprivation are hallmark features of the TME [102]. Under hypoxia, hypoxia-inducible factor (HIF) is induced, which plays a crucial role in the survival of tumor cells, as well as in tumor drug resistance [103]. YY1 directly binds to HIF-1a promoter and recruits RNA polymerase II and the histone acetyltransferase p300 to promote HIF-1a transcription under hypoxia. Moreover, YY1 binds to the oxygen-dependent degradation (ODD) domain of HIF-1a, blocks von Hippel–Lindau (VHL)-mediated ubiquitination, and promotes HIF-1a protein stabilization and nuclear translocation [6,104,105]. Consequently, YY1/HIF-1a upregulates VEGF expression and drives tumor vascularization to augment oxygen and nutrient perfusion. Also, YY1 upregulates the expression of VEGFA, resulting in the activation of the phosphorylation of VEGFR2, and triggers vascular endothelial morphogenesis and transmigration capacity. Subsequently, it promotes hepatocellular carcinoma (HCC) angiogenesis and resistance to bevacizumab. However, these newly formed blood vessels have abnormal structures and functions, leading to drug delivery failure [65,106,107]. Furthermore, YY1/HIF-1a promotes P-gp and medicine-related problems (MRPs) expression and increases drug efflux. Thereby, reduced intertumoral drug concentration contributes to the formation of multidrug resistance.

#### 2.5.1. YY1-Driven Metabolic Remodeling of the TME Promotes Drug Resistance

The hypoxic conditions and nutrient deficiency within the TME prompt tumor cells to undergo metabolic reprogramming, including upregulating glycolysis and altering nutrient transport and metabolic pathways, to meet the demands of proliferation and adapt to the harsh conditions [108,109]. Conversely, tumor cells alter metabolism patterns, remodel the extracellular matrix, and reshape the TME, exacerbating hypoxia and acidity, promoting angiogenesis, and creating a more suitable microenvironment for tumor cell survival, invasion, and metastasis. This interaction establishes a pathological ecosystem driving tumor progression and therapy resistance [17,110,111].

In multiple myeloma, YY1 promotes AKR1C3 expression and activates hedgehog signaling to promote glycolytic activity and lenalidomide resistance [96]. *KRAS* mutations occur in approximately 30–50% of patients and are a key determinant of non-responsiveness to chemotherapy in colorectal cancer (CRC) [112,113]. YY1 triggers EGFR/AKT/ERK pathway activation, enhancing *KRAS* membrane localization and reinforcing cetuximab resistance in *KRAS*-mutant CRC cells [114]. Polo like kinase 1 (PLK1) is another downstream effector of YY1, regulating cell cycle progression and metabolism. YY1-induced PLK1 expression elevates G6PD levels, activating the pentose phosphate pathway and increasing NADPH and glutathione (GSH) production, thereby driving tumor cells’ resistance to paclitaxel and cisplatin [115]. Furthermore, YY1 promotes lactate production through the upregulation of glycolytic enzymes, hexokinase 2 (HK2) and pyruvate dehydrogenase kinase 1 (PDK1). This leads to increased histone H3K18 lactylation (H3K18la), establishing a YY1–lactate–H3K18la positive loop, ultimately leading to cancer cell resistance to cisplatin [116].

Moreover, YY1 regulates lipid metabolism and affects drug resistance in the TME. Inhibitor of growth 5 (ING5) upregulated the expression of acetyl-CoA carboxylase 1 (ACC1) and ATP-citrate lyase (ACLY) and mediated tumor lipogenesis, which is involved in the malignant progression of cancers. Serum response factor (SRF)–YY1 complexes can bind to the promoter of ING5 and upregulate its transcription, thereby promoting lipid droplets’ (LDs) formation and increasing sorafenib resistance of HCC cells [117]. Galactosylcerebroside is known to be overexpressed on the surface of tumor cells and plays an important role in the inhibition of cellular adhesion and apoptosis. Lysosomal enzyme galactocerebrosidase (GALC) is responsible for the hydrolysis of galactosyl cerebroside. YY1 suppressed *GALC* gene transcription, consequently increasing galactosyl cerebroside accumulation and enhancing tumor cells’ resistance to doxorubicin [118]. Family with sequence similarity 60 member A (FAM60A) increases essential metabolic enzymes acyl-CoA synthetase long chain family (ACSL1/4) and glutathione peroxidase 4 (GPX4) as a key regulator. 

Additionally, the low amino acid signal activates the synergistic interaction between YY1 and yes-associated protein (YAP), activating the expression of the PH domain containing protein 6 (FGD6) gene and thereby forming the YY1–FGD6 axis. It can replenish the amino acid reserves within the cells, bypassing the dependence on free glutamine and maintaining metabolic balance. Studies have found that mTOR inhibitors can inhibit the growth of tumor regions rich in nutrients, but, in the central regions that rely on the YY1–FGD6 axis, the inhibitory function of mTOR inhibitors on cell growth is weakened [119].

#### 2.5.2. YY1-Mediated Immunomodulation in TME Remodeling Contributes to Drug Resistance

Tumor immunity is another critical factor in reshaping the TME. Immune cells within the TME, such as T cells (Tregs), B cells, NK cells, and macrophages, modulate tumor behavior by secreting cytokines, altering angiogenesis, and reshaping metabolic profiles [120,121]. Compelling evidence indicates that YY1 modulates tumor immunity by regulating immune checkpoint molecules, influencing T cell differentiation and function, and modulating myeloid-derived suppressor cell (MDSC) polarization [16,48]. YY1 directly transcriptionally activates PD-L1, LAG-3, and TIM3 transcription, which induce T cell exhaustion across various cancer types while enhancing tumor resistance to cisplatin and other chemotherapeutic agents [122]. Additionally, YY1 upregulates CD47 expression, inhibiting macrophage phagocytosis, facilitating tumor cell immune escape, and contributing to treatment resistance [123]. Moreover, YY1 also regulates immunosuppressive molecules, such as cytotoxic T-lymphocyte antigen 4 (CTLA-4), promoting the immunosuppressive function of regulatory T cells and inducing a tolerogenic phenotype in myeloid cells [123]. Regarding drug resistance mechanisms, YY1 not only mediates immune escape through immune checkpoints but also coordinately regulates tumor metabolism. For example, YY1 activates Hexokinase 2 (HK2)/pyruvate dehydrogenase kinase-1 (PDK1) to promote glycolysis, so that tumor cells generate excessive lactic acid, and impairs T cell function, expands regulatory T cells, and fosters an immunosuppressive microenvironment, thereby enhancing tumor cell resistance to both chemotherapy and immunotherapy.

The TME harbors multiple immunosuppressive components that collectively impair immune cells’ (including NK cells and T cells) function. The cytokine TGF-β suppresses T/B cell function and promotes T cell differentiation, while interleukin (IL-2/6/10) impairs antigen presentation and inhibits T/NK cell activity [124]. These immunosuppressive factors suppress anti-tumor immune responses through direct or indirect mechanisms that lead to tumor drug resistance [123,125,126]. YY1 inhibits the role of effector T cells by regulating the expression of TGF-β and IL-10. The functional inhibition of effector T cells leads to the escape of tumor cells from immune killing, which leads to resistance to immunotherapy. Researchers found that YY1 negatively regulates IL-2 (in collaboration with enhancer of zeste homolog 2 (EZH2) histone methyltransferase). As a type I cytokine, IL-2 plays a pivotal role in clonal expansion and the persistence of tumor-reactive T cells. However, YY1 represses *IL-2* gene activation, and repeat stimulation leads to CD^8+^ and CD^4+^ T cells showing markers of PD-1, LAG-3, and TIM3, which are all signs of T cell exhaustion [127,128].

EGFR-mutated patients notably secrete IL-32, which affects signaling pathways and biological processes linked to tyrosine kinase inhibitors’ (TKIs) sensitivity. YY1 upregulates the secretion of IL-32 in pericytes and subsequently activates the β5-integrin-Src-Akt (protein kinase B) pathway in EGFR-mutated lung cancer cells, thereby influencing sensitivity to TKIs [129]. Normally, the farnesoid X receptor (FXR) functions as a bile acid receptor that activates multidrug resistance-associated protein 2 (MRP2) expression. However, IL-18 (a pro-inflammatory cytokine) suppresses FXR activity while simultaneously activating the NF-κB/YY1 axis, contributing to the downregulation of MRP2 expression, therefore promoting cancer cells’ chemoresistance under the tumor microenvironment [130].

Interestingly, YY1 cooperates with HIF-1α to upregulate VEGF expression and facilitate the accumulation of immunosuppressive cells, including MDSCs and regulatory T cells, particularly in glioblastoma [41]. A study showed that YY1 could suppress anti-tumor immunity by enhancing HIF-1α stability in tumor-associated macrophages [131]. It also associates with small mother against decapentaplegic (SMAD)3/4 to inhibit forkhead box protein 3 (FOXP3) transcription, blocking the differentiation and function of T cells and further promoting immune suppression [132].

**Figure 5:**
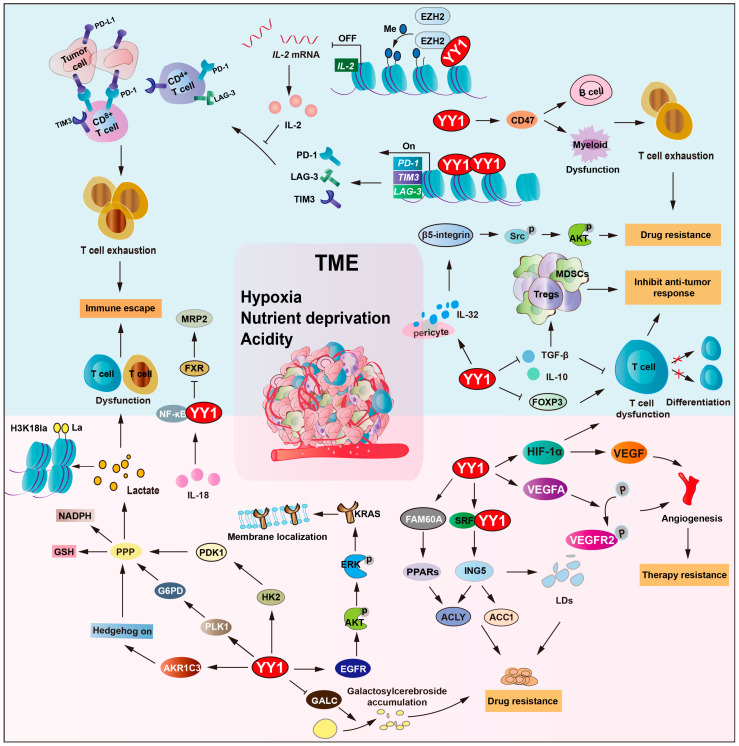
YY1 orchestrates TME remodeling to drive drug resistance. YY1 regulates glycolysis via HIF-1a and G6PD and creates an acidic hypoxic niche. It also upregulates CD47 to block macrophage phagocytosis and induces T cell exhaustion by repressing IL-2, GALC, TGF-b, IL-10, and FOXP3. Additionally, YY1 mediates TKI resistance through the IL-32/b-5-integrin/AKT pathway and activates the IL-18/NF-κB axis to downregulate MRP2, forming an inflammatory feedback loop. This metabolic–immune crosstalk enables tumor cells to escape immune surveillance and resist therapy-induced cell death, thereby developing drug resistance. Arrows are used to show activation; arrows terminated with a bar represent inhibition.

Overall, YY1 plays a key part in modulating tumor cell survival and chemoresistance within the TME by orchestrating important biological processes, such as metabolic reprogramming, immune evasion, and other cancer-associated pathways (Figure 5 and Table 3).

### 2.6. Role of YY1 in EMT-Mediated Tumor Drug Resistance

Epithelial–mesenchymal transition (EMT) enables epithelial cells to acquire invasive mesenchymal properties while fostering drug resistance through enhanced cellular plasticity and cancer stem cell traits [68,136,137]. More precisely, EMT core regulators (TWIST1, SNAIL, ZEB1) promote tumor cell migration by upregulating N-cadherin/vimentin [138,139,140]. These transcription factors concurrently activate stemness properties and drive therapy resistance (such as tamoxifen, platinum drugs, and paclitaxel) through exosomal signaling and RHOJ-mediated DNA damage repair [141,142,143].

**Figure 6:**
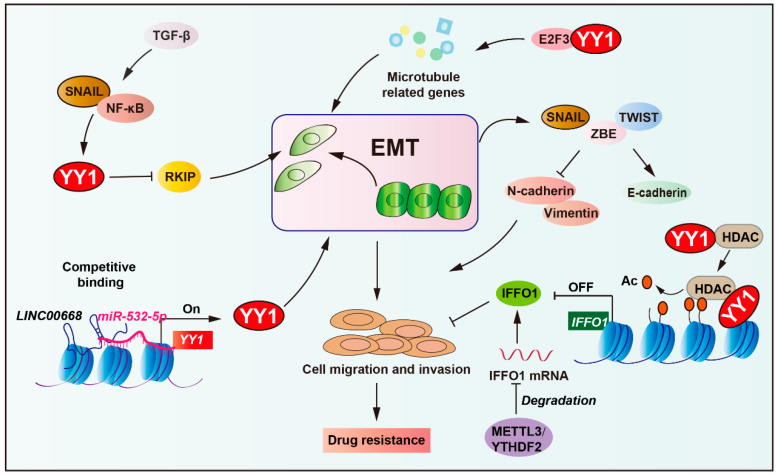
Role of YY1 in EMT-mediated tumor drug resistance. YY1, acting as a downstream effector of the NF-κB/SNAIL pathway, represses RKIP expression to drive tumor epithelial–mesenchymal transition (EMT). Concurrently, the long non-coding RNA *LINC00668* competitively binds to *miR-532-5p* to upregulate YY1, further enhancing EMT progression. Mechanistically, YY1 promotes tumor drug resistance via epigenetic regulation by recruiting HDAC5 to suppress IFFO1 for metastasis and cisplatin resistance. In addition, YY1 collaborates with E2F3 proteins to activate the expression of microtubule-related genes, thereby mediating drug resistance in tumors. This integrated network highlights YY1 as a key mediator linking EMT to therapeutic resistance. Arrows are used to show activation; arrows terminated with a bar represent inhibition.

Notably, EMT is regulated by multiple pathways (such as TGF-β, Wnt, RTK, and NF-κB), activating SNAIL/ZEB/TWIST to suppress E-cadherin and induce N-cadherin/vimentin expression, promoting cell migration and invasion. The tumor microenvironment (including hypoxia and inflammation) and epigenetic regulators (miRNAs and lncRNAs) critically modulate EMT dynamics, facilitating cancer dissemination and therapeutic resistance [68,144]. YY1 acts as a downstream effector in the NF-κB/SNAIL pathway, repressing RKIP expression to promote tumor EMT and treatment resistance, while NO donors downregulate YY1 to restore RKIP activity and reverse drug resistance (Figure 6) [145,146]. Additionally, YY1 is required for TGF-β-induced EMT in lung epithelial cells, acting through the NF-κB/SNAIL/RAF pathway dependent on chromatin remodeling [147].

Long non-coding RNAs, such as *LINC00668*, can promote EMT by regulating YY1 expression via competitive binding to *miR-532-5p*, thereby enhancing cell proliferation, migration, and invasion [148]. Moreover, YY1 cooperates with transcription factor 3 (E2F3) proteins to enhance the expression of microtubule-related genes, promote the ovarian cancer cell mesenchymal transition, and increase the resistance to paclitaxel and docetaxel [149]. In ovarian cancer, intermediate filament family orphan 1 (IFFO1) suppresses tumor metastasis and cisplatin resistance by inhibiting β-catenin nuclear translocation. Specifically, YY1 recruits histone deacetylase 5 (HDAC5) to the IFFO1 promoter, suppressing its expression, resulting in metastasis and cisplatin resistance [150]. Collectively, these findings identify YY1 as a critical nexus between EMT and treatment resistance, reinforcing its therapeutic potential in malignancies driven by EMT.

## 3. Targeting YY1 to Overcome Therapeutic Resistance in Cancer Treatment

### 3.1. Clinical Implications of Targeting YY1 in Overcoming Drug Resistance

As a multifunctional transcription factor, YY1 is abnormally highly expressed in most tumors and plays a key role in tumorigenesis and malignant progression. It could extensively regulate the core biological processes of tumors, such as cell growth, apoptosis, invasion, and metastasis, by directly binding to the promoter of target genes or interacting with other transcription factors [29,151]. Clinically, elevated YY1 expression is associated with advanced stage, metastasis, and treatment resistance, positioning it as both a valuable prognostic molecular marker and a therapeutic target [29].

Notably, YY1 is a central mediator of tumor drug resistance, driving multiple escape mechanisms, including ABC transporter upregulation, cancer stem cell maintenance, tumor microenvironment shaping, and pro-survival pathway activation. YY1 establishes a comprehensive defense system against anticancer agents. This central role in drug resistance pathways makes YY1 a promising therapeutic target, as its inhibition holds the potential to simultaneously dismantle multiple resistance mechanisms and resensitize refractory tumors to therapy [115,152,153]. Thus, YY1 represents a theranostic target capable of refining treatment strategies while improving clinical outcomes.

### 3.2. Combination Therapy Strategies Targeting YY1

Combination therapy has become a fundamental paradigm in modern oncology that overcomes the limitations of single-agent treatments. This multiple targeting strategy overcomes drug resistance, enhances efficacy, and reduces toxicity through synergistic effects. The rational combination of molecularly targeted agents with conventional therapies or immunotherapies provides comprehensive tumor control, overcoming heterogeneity while improving treatment outcomes. YY1’s central role in tumor progression and drug resistance makes it a promising target for combination therapy. Studies demonstrate that YY1 inhibitors promote the efficacy of therapeutic drugs, suggesting their potential in combination therapies, as shown in Table 4. Therefore, combining YY1 inhibitors with existing therapies (such as chemotherapy, immunotherapy, and targeted drugs) improves efficacy and overcomes resistance, offering a more effective strategy for cancer treatment [29,69].

#### 3.2.1. Combination with Chemotherapy Drugs

Chemotherapy still plays an important role in cancer drug treatment, leveraging cytotoxic agents to eliminate tumor cells. However, its efficacy is frequently hindered by insufficient drug accumulation in tumors and the formation of resistance-related mechanisms, leading to failed therapy and cancer recurrence [154,155,156]. Emerging evidence establishes YY1 as a key regulator of chemoresistance, coordinating multiple resistance pathways in various malignancies [43,84,157]. Chemotherapeutic drugs (5-FU, cisplatin, and paclitaxel) are limited by enhanced DNA repair capacity and the anti-apoptotic pathway in tumor cells, with YY1 mediating these processes. For example, in prostate cancer cells that are resistant to cisplatin, YY1 directly binds to the promoter region of the DNA repair gene SHILD, thereby increasing its expression and enhancing nucleotide excision repair efficiency. YY1 inhibitors, such as the small-molecule compound diethylamine NONOate (DETA NONOate), induce the s-nitrosation of YY1, leading to downregulation of BCL-XL expression and activating the apoptotic pathway, simultaneously increasing the sensitization of prostate carcinoma cells to cisplatin [158,159]. Resistance to doxorubicin is driven by P-gp-mediated drug efflux, which is upregulated under intermittent hypoxia via HIF-1α activation. YY1 enhances P-gp expression by stabilizing HIF-1α. A promising therapeutic strategy involves the combination of YY1 inhibitors (lercanidipine and amlodipine, calcium channel blockers with identified YY1 inhibitory activity) with doxorubicin. This combination therapy mechanistically targets multiple resistance mechanisms by disrupting the HIF-1α/P-gp axis while suppressing ERK/MAPK survival pathway and restoring doxorubicin sensitivity. The observed inhibition of tumor spheroid formation and the reversal of chemoresistance support its potential for cancer therapy [160,161,162,163]. Additionally, galiximab enhances non-Hodgkin lymphoma (NHL) cells’ sensitivity to cisplatin and TRAIL-induced apoptosis by inhibiting the YY1/BCL-XL pathway, and combination therapy improves anti-tumor efficacy [164].

Furthermore, nucleic-acid-based YY1 inhibitors (such as siRNAs, miRNAs, and CRISPR/Cas9) demonstrate effects when combined with conventional anti-tumor agents, effectively overcoming chemoresistance through targeted YY1 pathway inhibition. This combinatorial strategy has shown promising results in both in vitro and in vivo models of drug-resistant cancers. In a gastric cancer model, YY1 activates the transcription of NLRC5 and leads to cell invasion, migration, and resistance to 5-FU chemotherapy. Combined treatment with siRNA-YY1 and 5-FU decreased NLRC5 expression and increased tumor cell sensitivity to 5-FU-induced apoptosis [165]. Recently, research found that T7 ligand-functionalized exosomes (T7-exo) could enhance the delivery of cholesterol-modified siYY1. When combined with temozolomide, targeted knockdown of YY1 through T7-siYY1-exo overcomes chemotherapy resistance and suppresses tumor cell growth. T7-exosome binds to glioblastoma cells and improves the delivery of siYY1. Combined with temozolomide, targeting the knockdown of YY1 via T7-siYY1-exo overcomes chemotherapy resistance and inhibits the growth of GBM [42]. In addition, knocking out YY1 decreases the expression of TP73-AS1 and results in sensitivity to chemotherapeutic temozolomide in GBM [166]. These findings indicate that using nucleic acid technologies to inhibit YY1 and in combination with drugs might be potential tactics to reverse chemoradiotherapy resistance in tumors.

Therefore, the increasing development of YY1 inhibitors is a hopeful strategy for overcoming drug resistance and increasing therapeutic efficacy in combination with conventional chemotherapeutic drugs.

#### 3.2.2. Targeted Therapy Combination

Targeted therapies play a pivotal role in current cancer therapy, with high specificity to precisely engage with distinct molecular targets in tumor cells. By blocking oncogenic signaling pathways, inducing apoptosis, or inhibiting tumor angiogenesis, these agents significantly enhance tumor therapeutic effects. However, drug resistance still remains a critical obstacle limiting their clinical efficacy [29]. Typically, YY1 serves as a key regulator in activating bypass signaling pathways, thereby mediating resistance to targeted therapies. For example, DR5 is downregulated in TRAIL-resistant cells. Mechanistically, YY1 represses DR5 transcription by binding to its promoter, leading to resistance to TRAIL-induced apoptosis. DETA NONOate, as an inhibitor of YY1, restores the sensitivity of prostate cancer cells to TRAIL-induced apoptosis by inhibiting YY1 and increasing DR5 expression [52]. Additionally, DETA NONOate and rituximab inhibit YY1, resulting in the upregulation of FAS expression and sensitization to TRAIL-induced and FAS agonist antibody (CH-11) induced apoptosis in B-NHL cells [167].

Recent studies demonstrate that rituximab targeting YY1 reverses TRAIL resistance in B-NHL by suppressing YY1 expression and impairing its DNA-binding activity. This approach effectively sensitizes cells to TRAIL-induced apoptosis through robust activation of the type II mitochondrial pathway [168]. Furthermore, similar therapeutic effects were replicated using YY1-specific siRNA, validating YY1 as a critical molecular target for overcoming TRAIL resistance [51]. Notably, the combination therapy of rituximab and artesunate leads to significant downregulation of YY1 and activation of the FAS/CD95 pathway, accompanied by mitochondrial membrane potential disruption, which ultimately reduces drug resistance in NHL [169]. Recently, research revealed that ursolic acid (UA) exerts remarkable anti-tumor effects by reducing the binding of YY1 and serum response factor (SRF) to the promoter of ING5, thereby downregulating ING5 expression. This mechanism disrupts the ING5-mediated PI3K/Akt axis, reversing the chemoresistance of HCC cells to sorafenib [117].

Overall, understanding the regulation between YY1 and target therapy resistance is key to the development of innovative therapies. The combination of YY1 inhibitors to combat YY1-driven resistance may contribute to more effective cancer therapy.

#### 3.2.3. Immunotherapy Combination

Tumor immunotherapy, which activates the host immune system to attack tumors, has demonstrated durable response advantages. For instance, PD-1/PD-L1 inhibitors can induce long-term remission in melanoma and NSCLC. However, clinical benefits remain limited, with most patients showing primary resistance due to immunosuppressive microenvironments or impaired antigen presentation [123,170]. Additionally, acquired resistance frequently occurs through mechanisms like the upregulation of immune checkpoint factors or the accumulation of regulatory T cells [171]. YY1 promotes tumor immune escape by directly regulating immune checkpoint molecules, influencing T cell differentiation and function, and modulating the immune microenvironment [132,172]. NADPH oxidase 4 (NOX4) drives NSCLC resistance to EGFR-TKIs (gefitinib and osimertinib) and tumor immune escape through a YY1-dependent pathway. Specifically, NOX4 upregulation enhances YY1-mediated transcriptional activation of IL-8, consequently promoting PD-L1 expression and forming an immunosuppressive tumor microenvironment. Furthermore, the combination of GKT137831, which is a specific NOX4 inhibitor and reduces YY1 expression, and gefitinib synergistically promoted apoptosis and reduced resistance to both TKIs and immunotherapy [173]. The deubiquitinase ubiquitin-specific protease 7 (USP7) stabilizes YY1 expression by preventing its proteasomal degradation, thereby promoting HCC cell proliferation, migration, and therapy resistance. Isorhamnetin reverses this process by targeting USP7 to promote YY1 degradation. Notably, HMSN-ISO@ProA-PD-L1 Ab nanoparticles, which are dual-functional mesoporous silica nanoparticles combining isorhamnetin (ISO) and anti-PD-L1 antibody, demonstrate therapeutic effects, including specific tumor targeting coupled with significant modulation of the tumor immune microenvironment through MDSC reduction and enhanced T cell infiltration [69]. YY1 has been implicated in the regulation of PD-L1 expression through several signaling pathways involving complex crosstalk [16,105,153]. Therefore, targeting YY1 and its regulatory networks represents a promising combinatorial strategy to enhance anti-tumor immunity and overcome resistance to immune checkpoint inhibitors.

**Table 4:** Combination therapy with YY1 inhibitor.

YY1 Inhibitor	Combination Drug/Therapy	Mechanism	Outcome	Cancer	Current Status	Refs
Galiximab	Fludarabine	Inhibit survival/anti-apoptotic NF-κB pathway SNAIL/YY1	Inhibit tumor growth	DHBL	Preclinical research	[174]
Galiximab	Cisplatin	Inhibit NF-κB /SNAIL/YY1/BCL-XL circuit	Induce apoptosis	NHL	Preclinical research	[164]
*MiR-302b*	Cisplatin	Downregulate E2Fs and YY1 and then inhibit ITGA6	Affect DNA Repair and stemness and increase sensitivity to cisplatin	TNBC	Preclinical research	[175]
Lercanidipine, Amlodipine	Doxorubicin	Inhibit the YY1/ ERK/ TGF-β pathway	Inhibit the potential of cellular proliferation and spheroid formation	GC	Preclinical research	[163]
siYY1	5-FU	Inhibit YY1 and decrease NLRC5 expression	Increase tumor cell sensitivity to 5-FU-induced apoptosis	GC	Preclinical research	[165]
CRISPR/Cas9	Temozolomide	Knock out YY1 and decrease the expression of TP73-AS1	Decrease tumor aggressiveness and drug resistance	GBM	Preclinical research	[166]
l-NAME, DETA NONOate	Photodynamic therapy	Inhibit the NF-κB/ SNAIL/YY1/RKIP loop	Inhibit cell growth and EMT	PCa	Preclinical research	[176,177]
CA3	Osimertinib	Inhibit YAP1 and YY1 expression, activate the EGFR/MAPK axis	Induce autophagy	NSCLC	Preclinical research	[178]
siYY1	Oxaliplatin	Inhibit the YY1/GLUT3 axis	Inhibit glucose metabolism and cell proliferation, sensitize tumor cells to treatment	CRC	Preclinical research	[179]
*MiR-103a*	Radiotherapy	Inhibit NF-κB and YY1 expression	Decrease DNA damage repair and radioresistance	UBC	Preclinical research	[180]
DETA NONOate	Cisplatin	Inhibit YY1, BCL-XL	Sensitize the resistant tumor cells to apoptosis	PCa	Preclinical research	[159]
DETA NONOate	TRAIL	Inhibit YY1 and increase DR5 expression	Enhance sensitivity to TRAIL apoptosis	PCa	Preclinical research	[52]
DETA NONOate	Rituximab	Inhibit YY1 and upregulate FAS expression	Increase apoptosis	B-NHL	Preclinical research	[167]
*MiR-7-5p*	Temozolomide	Target the 3′-UTR of YY1 and decrease YY1 expression	Suppress cancer stemness and increase sensitivity to drugs	GBM	Preclinical research	[79]
Obatoclax	TRAIL	Inhibit YY1, upregulate DR5, and decrease MCL-1 expression	Reverse resistance to TRAIL-induced apoptosis	B-NHL	Preclinical research	[53]
Rituximab	TRAIL	Suppress YY1 expression and impair its DNA-binding activity	Sensitive to TRAIL-induced apoptosis	B-NHL	Preclinical research	[168]
Rituximab	Artesunate	Downregulate YY1 and activate the FAS/CD95 pathway	Increase apoptosis and sensitize to therapy	NHL	Preclinical research	[169]
Ursolic acid	Sorafenib	Reduce YY1 and disrupt the ING5-mediated PI3K/AKT signaling pathway	Inhibit tumorigenesis and reverse sorafenib resistance	HCC	Preclinical research	[117]
T7-siYY1-Exo	Temozolomide, irradiation	Decrease YY1 expression	Enhance chemoradiotherapy sensitivity and improve survival	GBM	Preclinical research	[42]
siYY1	Chemo radiotherapy	Downregulate YY1 and PLK1	Increase cell death	ESCC	Preclinical research	[115]
*MiR-411-3p*	Methotrexate	Inhibit YY1 expression	Motivate MTX’s cellular uptake and cytotoxic	ALL	Preclinical research	[181]
*MiR-7*	Cisplatin	Downregulate YY1 and KLF4	Inhibit proliferation and cell viability, decrease resistance	NHL	Preclinical research	[182]
*MiR-7*	CH11	Inhibit YY1 expression and increase FAS activity	Increase apoptosis	CRC	Preclinical research	[55]
*MiR-186*	Cisplatin	Degrade YY1 protein	Inhibit the formation of the tumor-initiating cell phenotype, reverse cisplatin resistance	GBM	Preclinical research	[78]
AMD3100	Cytarabine	Increase let-7a and inhibit YY1	Extend survival	AML	Phase I/II clinical trial stage	[46]
GKT137831	Gefitinib	Inhibit NOX4 expression and downregulate YY1	Increase cellular apoptosis	NSCLC	Preclinical research	[173]
Photodynamic	DR2	Inhibit the NF-κB/SNAIl/YY1/RKIP loop	Induce cell death	PCa	Preclinical research	[183]
Isorhamnetin	Anti-PD-L1 antibody	Target USP7 and promote YY1 ubiquitin-dependent degradation	Improve the tumor immune microenvironment and inhibit progression	HCC	Preclinical research	[69]
Vitamin D	Anti-PD-1 drug	Promote VDR interaction with YY1 and activate the transcription of VDBP	Improve anti-tumor efficacy	HCC	Preclinical research	[184]
Kenpaullone	Doxorubicin	Inhibit YY1, downregulate BCL-2	Increase apoptosis	NHL	Preclinical research	[185]

## 4. Challenges and Future Perspectives

YY1 acts as a regulator in the network of tumor drug resistance, exerting multifaceted resistance-related pathways, ranging from enhancing drug efflux through upregulation of ABC transporters to facilitating DNA damage repair. Moreover, YY1 reinforces therapeutic resistance by maintaining cancer stemness, driving EMT, and reprogramming tumor metabolism, creating a multifaceted defense against chemotherapy, targeted therapy, and immunotherapy [41,43,114]. Simultaneously, YY1 drives tumor cells’ drug-resistant phenotypes by regulating tumor metabolism and reshaping the immune microenvironment. Thus, YY1’s critical role as a regulator coordinating multiple drug resistance mechanisms highlights its potential as a therapeutic target for overcoming drug resistance in tumors.

Despite the promising therapeutic value of targeting YY1, significant challenges and limitations persist. YY1 exhibits dual roles in tumor biology, functioning as either an oncogenic driver or a tumor suppressor depending on tumor type, molecular context, and the tumor microenvironment. In the majority of tumors, YY1 acts as a prototypical oncogene, driving multiple hallmarks of cancer progression, including cell proliferation, invasion, metastasis, angiogenesis, and resistance to therapeutic interventions [186]. Notably, in pancreatic cancer (PDAC), YY1 is mainly a tumor suppressor, inhibiting cell proliferation, metastasis, invasion, and pancreatic clock regulation. Its high expression is associated with better outcomes in PDAC patients, and YY1 may indirectly inhibit PDAC by reducing the incidence of diabetes, providing a theoretical basis for the diagnosis and targeted therapy of pancreatic cancer [187,188]. Furthermore, YY1 exhibits dual regulatory functions in lung cancer, modulating both tumor-promoting and tumor-suppressing activities through diverse epigenetic modifications and signaling pathways [189]. This functional duality presents a therapeutic dilemma, as inhibition of YY1 might suppress oncogenic roles while potentially disrupting tumor-suppressive functions. Thus, targeting YY1 is infeasible, as inhibition may accelerate tumor growth in YY1-protective cancers. This context dependence requires more precise molecular stratification to guide therapeutic decision making regarding YY1 inhibition, activation, or pathway-selective regulation.

Additionally, tumor heterogeneity also affects the treatment outcome. The molecular mechanisms of this heterogeneity include epigenetic regulation, post-translational modifications, and protein interaction networks, among others [29]. Studies show that YY1 demonstrates significant heterogeneity in diverse tumor types. The molecular mechanisms of this heterogeneity include epigenetic regulation, post-translational modifications, and protein interaction networks, among others [190]. In pancreatic cancer neuroendocrine tumors (PNETs), the occurrence of YY1 gene mutations is different in insulinomas and non-insulinomas, and its protein expression is related to clinic outcomes [191]. Within the same tumor, both the single-cell level and microenvironmental factors can lead to the heterogeneity of YY1. For instance, in glioblastoma, YY1 is highly expressed in the stem-cell-like cell subpopulation, while its expression is lower in differentiated cells. During the cisplatin-resistant epithelial cell transformation process in bladder cancer, YY1 expression gradually increases, and histone lactylation modification (H3K18la) can activate its expression by enriching in the YY1 promoter region, resulting in cisplatin resistance [116]. Therefore, given the heterogeneity of YY1 in tumors, it is rather difficult to select a treatment plan.

A mountain of evidence supports the therapeutic value of targeting YY1 in drug-resistant malignancies. Small-molecule inhibitors, nucleic-acid-based agents (such as siRNAs, antisense oligonucleotides), and epigenetic drugs that modulate YY1 expression or activity have demonstrated promising results in preclinical models. Importantly, when combined with conventional chemotherapies, targeted therapies, or immunotherapies, these agents synergize to reverse resistance phenotypes, enhancing tumor cell vulnerability and improving treatment responses. This combinatorial strategy is especially attractive in the era of immuno-oncology. However, their effectiveness is often blunted by resistance mechanisms, many of which are reinforced by YY1 activity. For example, YY1-mediated immune evasion and CSC maintenance contribute to immunotherapy failure. Integrating YY1 inhibition into immunotherapy regimens may thus unlock durable anti-tumor immunity and expand the pool of patients who benefit from these treatments [192].

## 5. Conclusions

Collectively, YY1 serves as a central hub coordinating drug resistance mechanisms, making its therapeutic targeting a compelling strategy against refractory cancers. Therefore, future work should bridge mechanistic insights to clinical translation through network mapping, rational drug combinations, and biomarker development to harness YY1 inhibition’s full potential in precision oncology.

## Figures and Tables

**Table 1:** YY1 regulates cell death pathways in cancer drug resistance.

Anti-Cell Death Pathway	Target Gene	Regulation	Outcomes	Refs
NF-κB/SNAIl/YY1 axis	RKIP	Downregulates RKIP	Chemotherapy and immunotherapy	[47]
BCL-2 family/YY1	MCL-1	Upregulates MCL-1, downregulates caspases-3, -7, and -9	Anti-apoptosis	[50]
P53/YY1	DR5	YY1 and p53 synergistically inhibit DR5	Chemoresistance and TRAIL-based therapies	[54]
NF-κB/YY1/MCL-1 axis	DR5, MCL-1	Upregulates MCL-1 and downregulates DR5	Resistance to Obatoclax	[51]
RELA	BIM	The YY1/RELA complex inhibits the expression of BIM	FAS/FASL-mediated cell apoptosis	[44]
CRHR2/UCN2/*miR-7*/YY1 pathway	YY1	Inhibits YY1 and restores FAS/FasL-induced apoptosis	FAS/FASL-mediated cell apoptosis	[55]
YY1/KDM6A/TRKA	TRKA, NTRK1	Upregulates TRKA	Resistance to imatinib	[56]
YY1/KDM5C	YY1	KDM5C recruits YY1 to regulate the cell cycle	Broad-spectrum resistance	[57]
CRNDE/YY1/EGFR pathway	EGFR	Upregulates EGFR	Resistance to sorafenib	[58]
PART1/*miR-512-3p*/CHRAC1/YY1	CHRAC1	YY1 activates PART1 to inhibit *miR-512-3p*, upregulating CHRAC1	Resistance to cisplatin	[59]
YY1/*miR-135b*/BMAL1 pathway	BMAL1	YY1 activates *miR-135b*, inhibits BMAL1	Resistance to gemcitabine	[60]
TRIM34/YY1 pathway	TRIM34	Upregulates TRIM	Anti-ferroptosis	[61]
SIRT1/YY1/GPX4 axis	GPX4	Upregulates GPX4	Anti-ferroptosis	[62]
YY1/SLC7A11 pathway	SLC7A11	Upregulates SLC7A11	Resistance to ferroptosis-inducing agents erastin and RSL-3	[63]
YY1/SNX1/EGFR/PPARs-ACSL1/4 pathway	SNX1/EGFR/PPARs-ACSL1/4	Inhibits SNX1	Resistance to 5-FU	[64]

**Table 3:** YY1 reshapes the TME to drive drug resistance.

Pathway	Target Gene	Regulation	Resistance Type	Refs
YY1/VEGF/ VEGFR	VEGF/VEGFA	Upregulates HIF-1α	Resistance to bevacizumab	[133]
YY1/HIF-1α	HIF-1α	Upregulates VEGF, VEGFA expression	Resistance to multidrug treatment	[104]
Drug efflux pathway	P-gp, MRPs	Upregulates P-gp, MRPs’ expression	Resistance to multidrug treatment	[134]
Glycolysis–hedgehog pathway	AKR1C3	Promotes AKR1C3 expression, activates Hedgehog axis	Resistance to lenalidomide	[96]
EGFR/AKT/ERK-KRAS pathway	KRAS	Activates the EGFR/AKT/ERK pathway and promotes the localization of KRAS membranes	Resistance to cetuximab	[114]
PPP	PLK1, G6PD	Activates PPP	Resistance to paclitaxel and cisplatin	[115]
Glycolysis	HK2/PDK1	Upregulates HK2/PDK1, promotes the lactylation of H3K18 (H3K18la) positive feedback	Resistance to cisplatin	[116]
Lipid synthesis	ING5	The SRF–YY1 complex upregulates ING5 expression	Resistance to sorafenib	[117]
Sphingolipidosis unspecified	GALC	Inhibits GALC	Resistance to doxorubicin	[118]
Immune checkpoint pathways	PD-L1, LAG-3, TIM3	Upregulates PD-L1, LAG-3, and TIM3	Resistance to cisplatin	[122]
Immune escape	CD47	Upregulates CD47 and inhibits phagocytosis by macrophages	Resistance to immunotherapy	[123]
Immunosuppression	CTLA-4	Upregulates CTLA-4	Resistance to immunotherapy	[123]
Glycolysis/T cell	HK2/PDK1	Activates HK2/PDK1	Resistance to immunotherapy and chemotherapy	[116]
Immunosuppression	TGF-β, IL-10	Upregulates TGF-β and IL-10	Resistance to immunotherapy	[135]
Immune resistance	IL-2	YY1 and EZH2 synergistically inhibit IL-2	Resistance to immunotherapy	[127]
IL-32-β5-Integrin-Src-Akt	IL-32	Upregulates IL-32	Resistance to tyrosine kinase inhibitors	[129]
FXR-MRP2	FXR	IL-18 inhibits FXR activity and simultaneously activates the NF-κB/YY1 axis	Resistance to multidrug treatment	[130]
HIF-1α-VEGF	VEGF	Upregulates VEGF	Resistance to immunotherapy	[41]
SMAD3/4-FOXP3-T	FOXP3	YY1 binds to SMAD3/4 and inhibits FOXP3 transcription	Resistance to immunotherapy	[132]
Amino acid metabolism	FGD6	YY1 and YAP synergistically bind to the FGD6 promoter	Resistance to mTOR inhibitors	[119]

## Data Availability

No new data were created or analyzed in this study.

## Abbreviations

YY1	Yin Yang 1
MDR	mediating cancer multidrug resistance
CAFs	cancer-associated fibroblasts
SVEP1	pentraxin domain containing 1
TKIs	tyrosine kinase inhibitors
HDAC2	histone deacetylase 2
FEN1	flap endonuclease 1
LEN	lenalidomide
TME	tumor microenvironment
VEGF	vascular endothelial growth factor
PLK1	polo like kinase 1
HK2	hexokinase 2
ING5	inhibitor of growth 5
ACLY	ATP-citrate lyase
LDs	lipid droplets
FAM60A	family with sequence similarity 60 member A
GPX4	glutathione peroxidase 4
IL-32	interleukin 32
MRP2	multidrug resistance-associated protein 2
TRIM34	tripartite motif-containing protein 34
NO	nitric oxide
CRHR2	corticotropin-releasing-hormone-receptor-2
NTRK1	neurotrophic tropomyosin receptor kinase 1
IFFO1	intermediate filament family orphan 1
CHRAC1	chromatin accessibility complex subunit 1
DIOB	diosbulbin B
PARPi	polymerase inhibitors
ITGA6	integrin subunit alpha 6
IR	irradiation
PP2A	protein phosphatase 2A
SMAD	small mother against decapentaplegic
CTLA-4	cytotoxic T-lymphocyte antigen 4
USP7	ubiquitin-specific protease 7
VDR	vitamin D receptor
NHL	non-Hodgkin’s lymphoma
GBM	glioblastoma
BC	breast cancer
DHBL	disseminated human B-lymphoma
BH	bendamustine hydrochloride
ABCB	ATP-binding cassette subfamily B
TRKA	tropomyosin receptor kinase A
NOX4	NADPH oxidase 4
HR	homologous recombination
BER	base excision repair
G4s	guanine quadruplexes
SHLD1	shieldin component
AKR1C3	aldo-keto reductase family 1 member C3
BET	bromodomain and extraterminal
HIFs	hypoxia-inducible factor
GTPase	guanosine triphosphatase
PPP	pentose phosphate pathway
PDK1	pyruvate dehydrogenase kinase 1
ACC1	acetyl-CoA carboxylase 1
SRF	serum response factor
GALC	galactocerebrosidase
ACSL	acyl-CoA synthetase long chain family
IFN-γ	interferon-gamma
FXR	farnesoid X receptor
FOXP3	forkhead box protein P3
SIRT1	silent information regulator 1
FASL	fas ligand
UCN2	urocortin-2
KDM5C	lysine demethylase 5C
HDAC5	histone deacetylase 5
EGFR	epidermal growth factor receptor
CSCs	cancer stem cells
mCAFs	matrix-type cancer-associated fibroblasts
RKIP	raf kinase inhibitor protein
ISO	isorhamnetin
VDBP	vitamin D binding protein
MTX	methotrexate
PTEN	phosphatase and tensin homolog
*BRCA1*	breast-cancer susceptibility gene 1
VHL	von Hippel–Lindau
EZH2	enhancer of zeste homolog 2
MAPK	mitogen-activated protein kinases
UA	ursolic acid
